# Quantitative phenotyping of leaf margins in three dimensions, demonstrated on KNOTTED and TCP trangenics in *Arabidopsis*

**DOI:** 10.1093/jxb/eru062

**Published:** 2014-04-04

**Authors:** Shahaf Armon, Osnat Yanai, Naomi Ori, Eran Sharon

**Affiliations:** ^1^The Racah Institute of Physics, The Hebrew University, Jerusalem, Israel; ^2^The Robert Smith Institute of Plant Sciences and Genetics in Agriculture, The Hebrew University, Rehovot, Israel

**Keywords:** Arabidopsis, curvature, differential geometry, growth, leaf shape, lobes, waviness.

## Abstract

The geometry of leaf margins is an important shape characteristic that distinguishes among different leaf phenotypes. Current definitions of leaf shape are qualitative and do not allow quantification of differences in shape between phenotypes. This is especially true for leaves with some non-trivial three-dimensional (3D) configurations. Here we present a novel geometrical method novel geometrical methods to define, measure, and quantify waviness and lobiness of leaves. The method is based on obtaining the curve of the leaf rim from a 3D surface measurement and decomposing its local curvature vector into the normal and geodesic components. We suggest that leaf waviness is associated with oscillating normal curvature along the margins, while lobiness is associated with oscillating geodesic curvature. We provide a way to integrate these local measures into global waviness and lobiness quantities. Using these novel definitions, we analysed the changes in leaf shape of two *Arabidopsis* genotypes, either as a function of gene mis-expression induction level or as a function of time. These definitions and experimental methods open the way for a more quantitative study of the shape of leaves and other growing slender organs.

## Summary

Three-dimensional geometry of leaf margins is an important shape characteristic to distinguish different leaf phenotypes. Novel geometrical methods were defined, measured, and used to quantify waviness and lobiness of leaves.

## Introduction

Shaping via growth of soft tissues is one of the most fascinating processes in nature. It involves biological, chemical, and physical mechanisms, the interaction of which eventually leads to the resulting shape. The richness of morphological phenotypes in nature leads one to wonder how elaborate such growth processes need to be in order to achieve such variety. What are the mechanisms and principles that govern the shaping of soft, growing tissue and how are they regulated?

Biological studies have identified genetic pathways that are involved in the shaping of leaves during growth. These studies are often based on analysing the effect of genetic ‘perturbations’ on the resulting morphological phenotypes. As a result, shapes of leaves are known to be affected by various genetic, hormonal, and environmental perturbations (reviewed by Tsukaya, 2006; Efroni *et al.*, 2010). In recent years, we have seen a rising interest in developing quantitative methods for phenotyping of leaf surface shapes (Bensmihen *et al.*, 2008; Liu *et al.*, 2010) and developing advanced measurement tools for leaf growth kinematics (Rolland-Lagan *et al.*, 2005; Lee *et al.*, 2006; Wiese *et al.*, 2007; Remmler and Rolland-Lagan, 2012). Only in a few cases has the mechanism leading from the genetic perturbation to the final alteration of the leaf shape been determined to some level (Nath *et al.*, 2003).

The shape of leaf margins is an important characteristic in the overall leaf shape and is often used to define and classify specific phenotypes. Currently, leaf margin types are defined qualitatively to be, for example, entire (smooth), lobate (indented but not to the midline), or undulate (wavy). A quantitative description of leaf margin shapes is lacking.

Genetic studies have shown cases in which entire leaves become wavy or lobate, and vice versa, as a result of different genetic perturbations (e.g. Nath *et al.*, 2003; Palatnik *et al.*, 2003; Nikovics *et al.*, 2006; Blein *et al.*, 2008; Efroni *et al.*, 2008; Berger *et al.*, 2009; Blein *et al.*, 2013; Burko and Ori, 2013). As a consequence, it is generally believed that genetic processes ‘mark’ specific locations along the rim of the tissue to determine the boundary shape (e.g. Nikovics *et al.*, 2006).

A recent study (Coen *et al.*, 2004) suggested that, in order to describe the mechanisms leading from a genetic perturbation to the final alteration of a leaf shape, one needs to integrate the powerful tools of genetics and molecular biology with terminology and tools that are beyond conventional biology. Following that concept, the *characterization* of a shape should be based on proper *geometrical terminology* (and when dealing with curved bodies, on differential geometry). In addition, the *kinematics* of a shape should be analysed in terms of *mechanical stability*.

Recent works in the theory of elasticity have shown that thin flexible sheets that expand/shrink laterally but not uniformly can attain complex three-dimensional (3D) equilibrium configurations. In particular, enhanced growth towards a sheet’s edge can lead to equilibrium configurations with wavy edges or even multi-wave, fractal-like configurations (Sharon *et al.*, 2002; Audoly and Boudaoud, 2003; Marder *et al.*, 2003; Dervaux and Ben Amar, 2008; Efrati *et al.*, 2009). Experimental realization of such growing sheets, using environmentally responsive gels that swell differentially, has demonstrated these non-trivial, multiscale configurations (Klein *et al.*, 2007, 2011; Kim *et al.*, 2012). Some of the observed configurations highly resemble the shapes of some leaves.

In purely mechanical systems such as responsive gel sheets, the growth (or swelling) field is given, while the shape of the sheet is an outcome—selected by the equation of elasticity. Leaves, however, are not homogeneous elastic sheets. They have a complex internal structure, their mechanical properties are far more complicated than the assumption of linear elasticity, and biological processes change these properties due to different stimuli. Still, a leaf is a thin sheet of material that is in mechanical equilibrium, and it responds elastically to moderate, short-time deformations. It was thus suggested that spontaneous buckling and other mechanical principles could participate in the shaping of leaves and other plant organs (Green, 1999; Sharon *et al.*, 2002, 2004; Dumais, 2007; Liang and Mahadevan, 2009). In particular, enhanced growth of leaf margins relative to its center may be the origin of the wavy pattern of the rim. In such a case, wavelength would be set by the growth profile and the thickness, rather than by explicit genetic ‘marking’ of specific sites at early stages of leaf development.

Furthermore, lobiness can be considered another way to accommodate excess length of margins. A leaf with given lateral dimensions (length, radius, and width) can increase the length of its margins by letting them oscillate in plane (lobiness) and not just out of plane (waviness). The absence of geometrical definitions of margin shapes prevents quantitative analysis of the connection between the biological and geometrical aspects of leaf shaping.

In this study, we suggest quantitative measures to describe the margin shapes of growing leaves. Specifically, we provide geometrical definitions for the local amount of waviness and lobiness as the in-plane and out-of-plane curvatures along a leaf’s boundary. The combination of these two characteristics describe uniquely any possible leaf contour in 3D. In addition, we suggest definitions for the total value of waviness and lobiness in a given leaf.

**Fig. 1.**
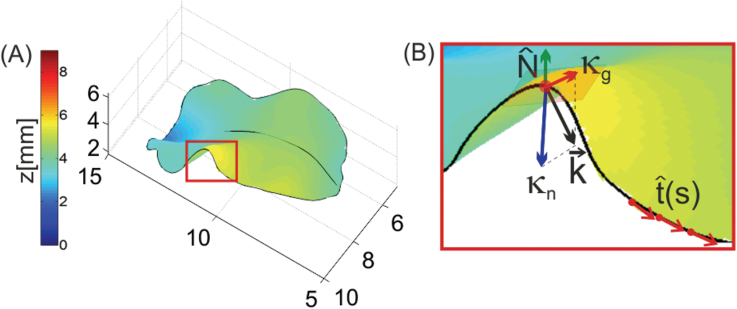
Decomposition of leaf margin local curvature. (A) A 3D measurement of an *Arabidopsis KAN>>miR319* leaf. Both surface topography (axes and colour bar in mm) and the edge curve (black line) are obtained. (B) At each point, *s*, along the margins we obtain the local tangent vector to the margins curve, $\hat{t}$ , and the local normal to the surface, $\hat{N}$ . The local curvature vector, $\vec{k}$ , is decomposed into its normal component$\;\kappa_{n}\;$ and geodesic component $\kappa_{g}$ . The local tangent plane is depicted as a shaded square. (This figure is available in colour at *JXB* online.)

The suggested phenotyping method is based on performing high-resolution measurements of the local curvature along a leaf edge in 3D (Fig. 1A). We performed these measurements and calculated the suggested quantities in wild-type *Arabidopsis* leaves, and compared them with two transgenic *Arabidopsis* lines with significantly altered leaf morphologies. In one case, a dose-dependent perturbation enabled us to characterize a range of phenotypic severity. In the other case, a perturbation to the maturation mechanism caused a phenotypic enhancement as the leaf grew. We thus present the evolution of lobiness and waviness as a function of the degree of the biological perturbation and as a function of time.

By using these well-defined quantitative geometrical measures, we might improve our understanding of the relationhips between biological activities, growth distribution, and organ shape.

## Materials and methods

### Geometrical definitions

The leaf edge is a curve in 3D. As such, the distinction between waviness and lobiness is sometimes not trivial. In order to distinguish between the two, we used the fact that the curve is part of the leaf surface. We suggest that edge waviness is associated with oscillating surface bending along the curve (a walker on the leaf edge goes up and down with the surface), while lobiness is associated with oscillating ‘turning rates’ of the curve (the walker turns right or left on the surface). We can thus locally decompose the curvature, $\vec{k}$ , of the edge curve into two components, one that is locally perpendicular to the surface and one that is locally tangential to the surface. In the terminology of the theory of surfaces, these are the normal and geodesic curvatures, $\kappa_{n}$ and $\kappa_{g}$ , respectively (see O’Neill, 2006, for definitions of geometrical quantities) (Fig. 1B). These two basic local quantities properly represent the geometry of leaf margins. Plotting $\kappa_{n}$ and $\kappa_{g}$ as functions of the arc-length parameter along the margins, *s*, provides quantitative information about the leaf shape. For example (see also Supplementary Fig. S1 at *JXB* online): a flat circular leaf of radius *r* would have $\kappa_{n}=0$ and $\kappa_{g}=\frac{1}{r}$ at every point. A circular dome-like leaf would have $\kappa_{n}=Const\;\neq 0$ and $\kappa_{g}=Const$ . An elliptical flat leaf would have $\kappa_{n}=0$ , while $\kappa_{g}$ would be small along the long axis margins and would increase towards the pointy tips. The proposed local decomposition can handle surfaces that consist of non-trivial combinations of $\kappa_{n}$ and $\kappa_{g}$ .

Having these local measures of in-plane and out-of-plane curvatures, we can propose measures for the total magnitude of waviness and lobiness over some interval of the margins, or over an entire leaf perimeter. This is obtained by proper and separate integrations of the oscillations of $\kappa_{g}(s)$ and $\kappa_{n}(s)$ . We use the ratio, *R*, between a leaf’s perimeter and diameter as a measure for the total excess length of leaf margins.

### Leaf measurements and analysis

The suggested measurement and analysis methods, which are applicable for any surface boundary, consist of the following steps:

- The 3D leaf surface is measured using an optical profilometer (MiniconScan 3000; Optimet) that scans the surface and calculates its topography z(x,y) using the scattered light pattern. The tool provides high-resolution measurements (50 μm in *x*–*y*, 5 μm in *z*) over an entire leaf area (typically 3–20mm long). For more details see Supplementary Information S2 at *JXB* online.
- The surface function z(x,y) is smoothed using a spline algorithm (Matlab7 Toolbox) in order to eliminate the effects of trichomes and other spikes. The perimeter curve is obtained using an edge detection algorithm (see also Supplementary Information S2).
- The excess length ratio is calculated as R=p/d , where p  is the leaf 3D perimeter and *d* is the length of the leaf long axis_,_ both measured in 3D.
- The perimeter curve is divided into segments of equal length, ds . The curve is now denoted as $\vec{\gamma }(s)$ . For each point, we compute the local tangent vector, $\vec{t}\left(s\right)=\frac{d\vec{\gamma }}{ds}$ and the local curvature vector, $\vec{k}(s)=\frac{d^{2}\vec{\gamma }}{ds^{2}}$ (Fig. 1B).
- The local normal vector to the surface along the contour is computed by:

$$\hat{N}\left(x,y\right)=\frac{-\frac{dz}{dx}\hat{x}-\frac{dz}{dy}\hat{y}+\hat{z}}{\sqrt{1+\frac{dz}{dx}+\frac{dz}{dy}\;}}$$

- where a ‘^’ sign denotes that a vector is normalized to have a magnitude of 1 (Fig. 1B).
- $\kappa_{n}(s)$ and $\kappa_{g}(s)$ are calculated as projections of $\vec{k}(s)$ onto $\hat{N}(s)$ and on the tangent plane (Fig. 1B):

$$\kappa_{n}\left(s\right)=\vec{k}\cdot \hat{N}$$

$$\kappa_{g}\left(s\right)=\vec{k}\cdot (\hat{N}\times \hat{t})$$

- In order to associate a single value for the total waviness and lobiness of a 3D curve, a synthetic planar curve is constructed from each of the signals $\kappa_{n}\left(s\right),\kappa_{g}\left(s\right)$ separately. These theoretical curves contain only the chosen curvature component. This is done by solving the two following equations independently:

$$\frac{d{\hat{\tau }}_{l}}{ds}=\left(\kappa_{g}-<\kappa_{g}>\right)\hat{n}$$ (1)

$$\frac{d{\hat{\tau }}_{w}}{ds}=\left(\kappa_{n}-<\kappa_{n}>\right)\hat{n}$$ (2)

- where$\;{\hat{\tau }}_{l/w}$ is the tangent vector of the planar curve, $\frac{d{\hat{\tau }}_{l/w}}{ds}$ is its two-dimensional curvature, and $\hat{n}$ is the normal vector to the planar curve (see illustrations in Supplementary Fig. S1). By integrating these equations, we extract the tangent vector ${\hat{\tau }}_{l/w}\;$ and hence obtain two curves in two dimensions. These curves rotate in the plane only with the relevant curvature component: $\left(\kappa_{g}-\kappa_{g}\right)$ or $\left(\kappa_{n}-\kappa_{n}\right)$ . The mean of the curvature component over the full measured interval, $<\kappa_{l/w}>$ , is subtracted, as waviness and lobiness are the fluctuations from that global contour curvature (for any flat closed curve we have$\;<\kappa_{g}>=2\pi$ . If the surface is not flat, $<\kappa_{n}>$ may be non-zero even without oscillations, for example on a contour of half a sphere $<\kappa_{n}>=2\pi$ ).
- Our suggested definitions for the total value of lobbiness, *l*, and waviness, *w*, are:

$$l=\frac{p-\left|\int_{0}^{p}{\hat{\tau }}_{l}ds\right|}{p}$$ (3)

$$w=\frac{p-\left|\int_{0}^{p}{\hat{\tau }}_{w}ds\right|}{p}$$ (4)

- where p  is the length of each planar curve, which is identical to the length of the original 3D leaf perimeter curve. Each of the vectorial integrals expresses the vector connecting the beginning and end points of each planar curve. We take the length of this vector (norm of a vector $\vec{a}$ is denoted by $\left|\vec{a}\right|$ ). *l* and *w* express the length added to that straight line due to the oscillations in the plane (after normalization by *p*). Therefore, *l* and *w* represent the (normalized) length added to the margins due to lobiness and waviness, respectively (see also Supplementary Information S3 at *JXB* online). The measures *w* and l  are non-negative numbers that do not depend on the length of the curve that is being measured. Also, the two quantities are independent and do not add up to any constant (a total excess length can be defined as $\;e.l.=\sqrt{w^{2}+l^{2}}$ . It represents the relative length added to the margins due to both lobiness and waviness. *e.l.* may attain any none negative value). In addition, Equations (3) and (4) are invariant to rescaling (swelling/shrinking of the whole leaf) and are not sensitive to small fluctuations in the curvature measurement. It is important to note, however, that other definitions of global waviness and lobbiness (which are based on $\kappa_{g}$ and $\kappa_{n}$ ) are possible.

### Transgenic plants preparation

**Fig. 2.**
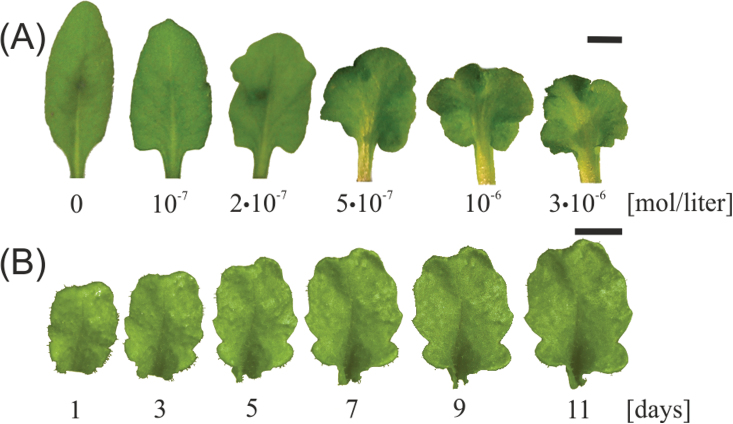
*Arabidopsis* leaf shape phenotypes. (A) *35S:kn1-GR* leaves, sprayed with increasing dexamethasone concentrations (as indicated). Bar, 5mm. (B) A *KAN>>miR319* leaf at different ages. Bar, 5mm. (This figure is available in colour at *JXB* online.)

We prepared two transgenic *Arabidopsis* genotypes, in order to quantify the effect of KNOX and TCP transcription factor activity on the margin shapes. The first genotype was *35S:kn1-GR* (Fig. 2A), which expresses a fusion of the maize KNOTTED1 (KN1) protein with the steroid-binding domain of the glucocorticoid receptor (GR), under the control of the cauliflower mosaic virus 35S promoter. In this genotype, the KN1–GR fusion protein was active only upon application of dexamethasone (DEX). Induction of KN1–GR activity by DEX resulted in dramatic alterations of leaf shape in a dose-dependent manner: the normally entire, oval leaves became round, wavy, and lobate (Hay *et al.*, 2003). Here we sprayed different plants with DEX solution at concentrations of 0.1, 0.2, 0.5, and 1 μmol l^–1^. Entire seedlings were sprayed 10 days after sowing. Plants were grown in short-day conditions. Measurements were performed 2 weeks from spraying, on leaf #5. Nine leaves of each DEX concentration were measured.

The second genotype was *KANADI1>>miR319* (*KAN1>> miR319*) (Fig. 2B), which expresses miR319, a negative regulator of five CIN-TCP genes, under the control of the KANADI1 promoter, using a *trans*-activation system (Efroni *et al.*, 2008). TCP genes encode a family of plant-specific transcription factors that are involved in controlling the balance of plant growth and differentiation. CIN-TCPs are thought to promote leaf maturation and differentiation (Ori *et al.*, 2007; Efroni *et al.*, 2008; Martín-Trillo and Cubas, 2010). This genotype exhibits a postponed maturation process and therefore long-lasting growth, especially along leaf margins (Efroni *et al.*, 2008). The result is a leaf with a ruffled edge (Fig. 1B). We grew such plants in short-day conditions, and followed the margins shape of leaf #5, starting 3 weeks from sowing. The leaf was measured every 1h.

## Results

**Fig. 3.**
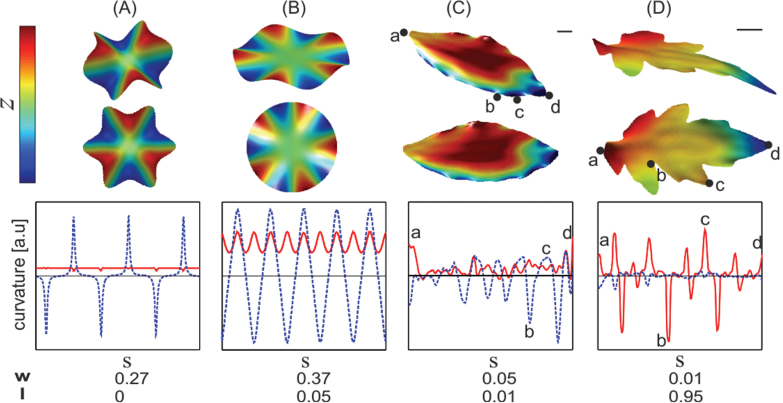
Waviness and lobiness of synthetic surfaces and common leaves. The images (top panels) show the surface topography from two different viewpoints. Surface colour represents the *z* coordinate in arbitrary units. The plots (bottom panels) display local curvature components $\kappa_{n}$ (dashed blue) and $\kappa_{g}\;$ (solid red) as functions of arc-length coordinate along the perimeter, *s*. (A) A mathematical wavy surface of constant radius (measured along the surface). (B) A mathematical wavy surface with oscillating radius. (C) A wavy *Pittosporum* leaf. (D) A flat and lobate tomato leaflet. Bars, 5mm. The measured values of *w* and *l* (see main text for definitions) are shown below the plots. (This figure is available in colour at *JXB* online.)

As a first step, we applied the analysis to mathematical surfaces (Fig. 3A, B) as well as to some common leaves (Fig. 3C, D). By looking at the artificial surfaces, it is difficult to determine whether a wavy surface is entire or lobate. In fact, due to the surface’s waviness, a top view might lead one to the wrong conclusions: a disc of constant radius might appear lobate (Fig. 3A, top panel), while a disc with varying radius might look like a circle (Fig. 3B, top panel). Our analysis captured the real geometry of the surface’s margins, yielding $\kappa_{g}\left(s\right)=Const$ in the first case and an oscillating geodesic curvature in the second case (Fig. 3A, B, bottom panels). The values of *l* quantitatively reflect the different lobiness of the surfaces.

The analysis was then applied to prototypes of wavy and lobate leaves. The leaf of *Pittosporum eugenioides* (lemonwood) is wavy. The normal curvature along its margins oscillates in correlation with the observed waviness of the boundary (Fig. 3C). The measurement showed the up–down asymmetry of the waviness: the upper bends of the waves were less curved than the bottom ones. The analysis revealed small oscillations of the geodesic curvature, indicating that the leaf was not perfectly entire. In addition, the average of $\kappa_{g}(s)$ along the leaf blade was small, with two sharp peaks. This indicated the overall elliptic shape of the leaf and its pointy tip/base. In contrast, the tomato leaflet is lobate, i.e. the geodesic curvature along its margins oscillates sharply (Fig. 3D). The locations of lobes/sinuses and their sharpness were clearly represented by $\kappa_{g}(s)$ . The tomato leaf was nearly flat: $\kappa_{n}(s)$ was very close to zero. The global measures, *w* and *l*, of the leaves clearly represented their wavy/lobate nature.

We next measured and analysed the changes in margin shape of the transgenic *Arabidopsis* genotypes.

### *35S:Kn1-GR*: static measurements

**Fig. 4.**
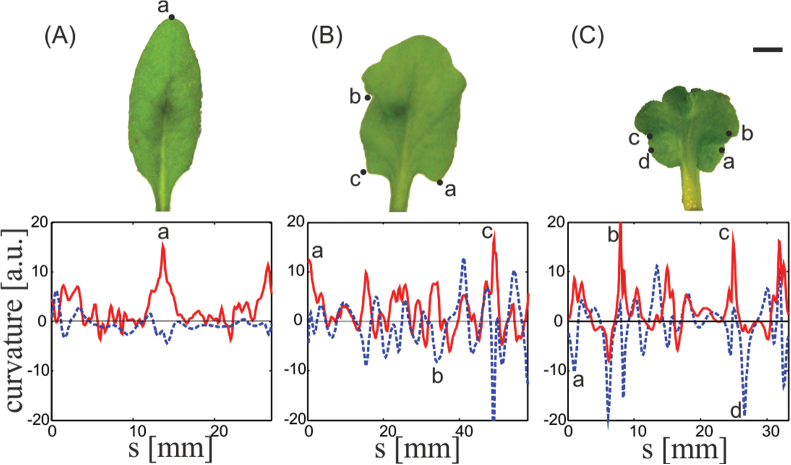
Waviness and lobiness of *Arabidopsis 35S:Kn1-GR* leaves. (A–C) Leaves treated with DEX at different concentrations (from left to right: 0, 2×10^–7^, and 1×10^–6^ mol l^–1^). The variation in the geometry of leaf margins is captured quantitatively by $\kappa_{n}(s)$ (dashed blue) and$\;\kappa_{g}(s)$ (solid red). The letters on the top and bottom panels show the close correlations between specific features on the leaf and the local measurements of $\kappa_{g}(s)$ and$\;\kappa_{n}(s)$ . Bar, 5mm. (This figure is available in colour at *JXB* online.)

**Fig. 5.**
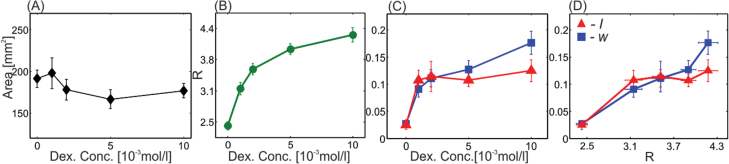
Changes in leaf geometry of *Arabidopsis 35S:Kn1-GR*. (A) The overall leaf area as a function of DEX concentration. (B) The ratio, R , between leaf perimeter and diameter as a function of DEX concentration. (C) The waviness, w (squares), and lobiness, l (triangles), as functions of DEX concentration. (D) The waviness and lobiness in (C) as functions of *R*. Each data point is the averaged result over nine leaves. (This figure is available in colour at *JXB* online.)

With increasing induction level (DEX concentration), the leaves became more circular, wavy, and lobate. Measurements of $\kappa_{n}(s)$ and $\kappa_{g}(s)$ showed the increase in the fluctuations of these two local quantities (Fig. 4). As mentioned above, waviness and lobiness of leaf margins are connected to other geometrical properties of the surface. In particular, they are associated with, and possibly result from, enhanced growth along the margins. Indeed, measuring the ratio, *R*, between the leaf perimeter and diameter showed a monotonic increase with the induction level (Fig. 5B). Wild-type leaves were elliptic and dome-like, having an average R=0.75π . In the highest manipulation degrees, we found R=1.3π (Note that a flat circular leaf would have  R=π). The increase in *R* was monotonic, while the leaf area was nearly constant (Fig. 5A). Both global waviness and lobiness increased sharply at low DEX concentrations. This trend flattened at higher DEX concentrations (Fig. 5C). The increase in *l* and *w* with *R* was more monotonic (Fig. 5D). This might indicate that a *primary* geometrical outcome of *kn1* overexpression is an increase in margin length, while lobbiness and waviness are outcomes of this increase.

### *KAN1>>miR319*: dynamic measurements

**Fig. 6.**
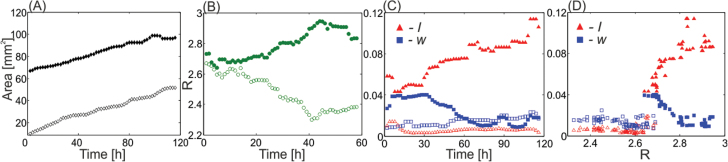
Changes in a single leaf marginal shape during growth: comparison between wild-type (open symbols) and *KAN1>>miR319* (filled symbols) leaves. (A) Leaf areas versus time. (B) Excess length ratios, *R*, versus time (C) The waviness, w (squares), and lobiness, l (triangles), as functions of time. (D) The waviness and lobiness as in (C) as functions of *R*. (This figure is available in colour at *JXB* online.)

Leaves of this genotype have complicated shapes. It is possible that a leaf is monotonically approaching its final shape that was pre-defined by the genetic perturbation in early leaf stages. Alternatively, the complex shapes could emerge from a dynamic process, in which growth regulation is perturbed but no ‘final’ leaf shape is defined. To address this question, we measured the evolution of the 3D shape of a growing *KAN1>>miR319* leaf over time,  t , during 5 days. Similar measurements were performed on a wild-type leaf. Both genotypes were found to grow at comparable growth rates (Fig. 6A); however, the temporal evolution of leaf *shape* was qualitatively different. Although the wild-type leaf increased its area considerably (Fig. 6A), its excess length ratio, *R*, was decreasing (Fig. 6B), while both lobiness and waviness remained very low (Fig. 6C). This indicated that the leaf became more concave (dome-like), keeping its margins entire. In contrast, the margins of *KAN1>>miR319* became relatively longer: *R* increased with time. In addition, leaf waviness was mostly decreasing from some non-negligible value, while lobiness was increasing. This indicated that the leaf, which already had a non-trivial margin shape, was becoming more flat and lobate during the measured time interval. The measured variations of *w* and *l* with time were preserved when the data were plotted versus *R* (Fig. 6D).

## Discussion

We introduced the normal and geodesic curvatures as well-defined measures for leaf margins geometry. The normal curvature captures the contour oscillations normal to the leaf surface (i.e. perpendicular to the local tangent plane) and the geodesic curvature captures the oscillations within the leaf surface (the ‘turning rate’ in the local tangent plane). Deviations of these quantities from their mean represent local waviness (normal curvature) and lobiness (geodesic curvature). Global measures for waviness/lobbiness of a leaf were developed (Equations 3 and 4). We demonstrated the use of these quantifications on *Arabidopsis* leaves with different levels of *kn1* mis-expression. These measurements suggested a scenario in which the specific gene mis-expression led to enhanced growth of the leaf margins. Waviness and lobiness may be outcomes of this enhanced growth, being two geometrical ways of accommodating the extra perimeter length.

Comparison of the temporal evolution of leaf shape between *Arabidopsis KAN1>>miR319* and wild-type plants showed that the transgenic leaf changed its shape dramatically during growth, much more than the wild-type leaf. Following an initial increase, its waviness decreased with time, while its lobiness underwent the opposite trends at the same time. Thus, it seems that *KAN1>>miR319* leaves do not grow ‘towards’ some pre-defined final leaf shape but evolve via a dynamic process. It is plausible that the main effect of the genetic manipulation is a perturbation to some growth-regulating mechanism. This perturbation results in a less coherent growth, which is manifested by significant temporal variations in leaf shape.

The geometrical quantities we have presented allow quantification of variations in leaf margin shapes induced by biological or environmental conditions. Moreover, they allow us to pose questions of a geometric nature about the origin of such phenotypes. For example, is the evolution of leaf geometry self-similar in time (include only rescaling of a given shape)? What is the wavelength of the observed oscillatory shape? What is the specific geometrical profile of the lobe/wave: is the curvature constant (sinusoidal edge) or pointy (serrations)? Is the creation of the two properties, waviness and lobiness, correlated in space or in time? The answers to such questions can shed light on the underlying principles that govern leaf shaping.

In particular, leaf marginal shape may provide indications for the underlying leaf growth profile. For example, marginal waviness can appear even under growth that is uniform along the edge, if it is enhanced compared with the interior parts of the leaf. In contrast, lobiness in its proposed definition requires non-uniform growth along the margins. Hence, quantitative measurements of leaf shape for various genotypes may provide information on the growth distribution (growth fields) within the leaves. Examination of the relationship between leaf margin shape and its surface growth field is left for future works.

## Supplementary data

Supplementary data are available at *JXB* online.

Supplementary Fig. S1. Examples of curvature analysis on simple surfaces.

Supplementary Information S2. Surface measurements and processing.

Supplementary Information S3. Global measures computation.

Supplementary Data

## Abbreviations:

3D: three-dimensional
DEX: dexamethasone.
