# Supplementary material for: Quantitative phenotyping of leaf margins in three dimensions, demonstrated on KNOTTED and TCP trangenics in Arabidopsis
Source: J Exp Bot. 2014 Apr 4;65(8):2071–7. doi: 10.1093/jxb/eru062 (PMC3991741; doi:10.1093/jxb/eru062)
Supplement: Supplementary Data [file supp_65_8_2071__index.html]

Quantitative phenotyping of leaf margins in three dimensions, demonstrated on KNOTTED and TCP trangenics in Arabidopsis — Quantitative phenotyping of leaf margins in three dimensions, demonstrated on KNOTTED and TCP trangenics in Arabidopsis — Supplementary Data 

# Quantitative phenotyping of leaf margins in three dimensions, demonstrated on KNOTTED and TCP trangenics in *Arabidopsis*

## Supplementary Data

Data files

**Files in this Data Supplement:**

- Supplementary Data - Supplementary Data
