# Supplementary material for: Quantitative phenotyping of leaf margins in three dimensions, demonstrated on KNOTTED and TCP trangenics in Arabidopsis
Source: J Exp Bot. 2014 Apr 4;65(8):2071–7. doi: 10.1093/jxb/eru062 (PMC3991741; doi:10.1093/jxb/eru062)
Supplement: Supplementary Data [file supp_eru062_jexbot109777_file001.pdf]

Quantitative Phenotyping of Leaf Margins in 3D,

Demonstrated on KNOTTED and TCP trangenics in Arabidopsis

Supplementary Online Material

Authors: Shahaf Armon, Osnat Yanai, Naomi Ori and Eran Sharon

SI1 - Examples of curvature analysis on simple surfaces

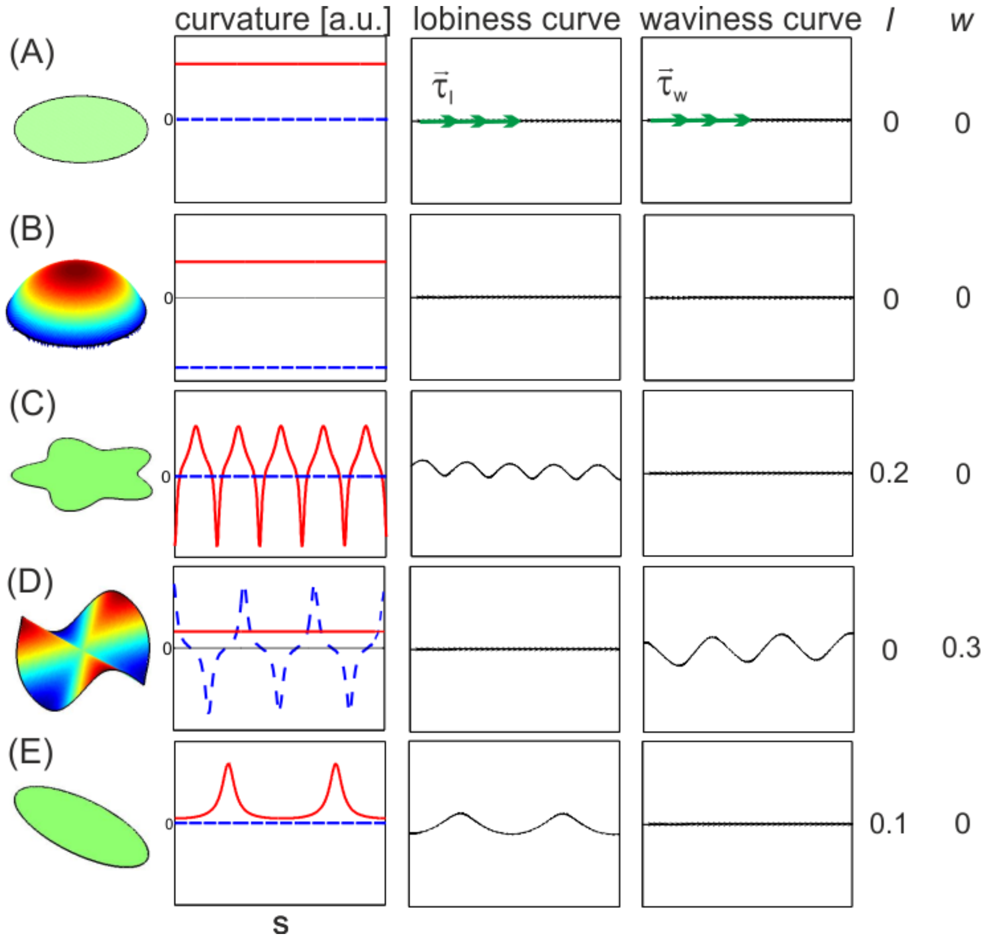

Fig. S1: Margins shape analysis on simple mathematical surfaces.

We apply our margins-shape analysis on simple mathematical surfaces (Fig. S1). The five surfaces chosen are: (A) flat circle (B) half a sphere (C) flat lobed surface (D) wavy surface with constant radius (E) flat ellipse. Color represents the surface height ($z(x,y)$). A perimeter curve is marked by a solid black line. The left most column shows the components of the 3D curvature of the perimeter curve: the Geodesic curvature (κ_g, solid red line) and the normal curvature (κ_n, dashed blue line). The middle and the right columns show the curves that represent the perimeter's lobiness and waviness respectively. Each of these planar curves contains only the scalar curvature $\kappa_g - < \kappa_g >$ or $\kappa_n - < \kappa_n >$. The tangent vector of

these curves is denoted by τ_w or τ_l (local example of such tangent vectors are illustrated) . The total lobiness and waviness are calculated by comparing the 2D curve length to the distance between its end points. When such a 2D curve is straight, the relevant quantity (waviness or lobiness) equals zero. When it oscillates, the global measure represents the added length by those oscillations.

S2 – Surface measurements and processing

The 3D leaf surface is measured using an optical profilometer (MiniconScan 3000, by Optimet). It is a ready-made tool that scans 3D surfaces using a red laser beam and calculates the surface height $z(x,y)$ using conical holography (Enguita et al., 2005). We use the tool for high resolution scans (50 μm in x-y, 5 μm in z) on a large field of view (up to $2 \cdot 2$ cm).

For growing leaf measurements, a living plant is placed in its pot on the tool's stage. The leaf is periodically scanned in constant time gaps (Microsoft Task Scheduler). The lighting is preset for short-day conditions (8 hours of light per day). The scanned leaf is kept free of any external constrains. All leaf scans in this work were programmed to last less than 10 minutes, in order to neglect shape changes during the scans.

The measured surface is smoothed using a smoothing spline algorithm in order to eliminate trichomes and noise. The exterior boundary of the leaf is traced in order to extract the perimeter curve x_i, y_i, z_i, i being an increasing index along the leaf edge. The curve is smoothed using low pass and diffusive filters. Then, data points are re-distributed to be equally spaced along the curve in order to obtain the curve in arc length parameterization $x(s), y(s), z(s)$ with Δs=0.05mm (~500 points in each leaf). Curvature is calculated by taking simple derivatives of the smoothed perimeter curve in the given parameterization.

S3 – Global measures computation

Given the two curvature components $\kappa_g(s), \kappa_n(s)$, the planar curves are obtained as follows: First, we eliminate the mean of the signals: $\kappa_g - <\kappa_g>; \kappa_n - <\kappa_n>$. We then obtain the 2D tangent vectors $\hat{\tau}_l(s)$, $\hat{\tau}_w(s)$ by solving the equations

$$\frac{d\hat{\tau}_l}{ds} = (\kappa_g - <\kappa_g>)\hat{n}$$

$$\frac{d\hat{\tau}_w}{ds} = (\kappa_n - <\kappa_n>)\hat{n}$$

where $\hat{\tau}_{l/w}$ is the tangent vector of the planar curve, and $\hat{n}$ is the normal vector to the planar curve.

To do so, we use Matlab7 differential equation toolbox and use the arbitrary initial conditions $\hat{\tau}_l(s=0)=\hat{x}$; $\hat{\tau}_w(s=0)=\hat{x}$, $\hat{n}(s=0)=\hat{y}$. Keeping the length of each segment, Δs, as in the original 3D curve, we can draw the curve in the plane (see Fig. S1, middle and right columns).

In order to calculate the global scalar quantities, we numerically integrate the vectors $\hat{\tau}_l, \hat{\tau}_w$ and compute the norm of the resultant vector which is the distance between end points. p is the full length of the 2D curve, which is identical to the original 3D leaf perimeter. Using these quantities, the global lobiness and waviness are defined by:

$$l = 1 - \frac{1}{p}\left|\int_0^p \hat{\tau}_l ds\right|$$

$$w = 1 - \frac{1}{p}\left|\int_0^p \hat{\tau}_w ds\right|$$
